# Microstructure and Superconducting Properties of Bi-2223 Synthesized via Co-Precipitation Method: Effects of Graphene Nanoparticle Addition

**DOI:** 10.3390/nano13152197

**Published:** 2023-07-28

**Authors:** Siti Nabilah Abdullah, Mohd Mustafa Awang Kechik, Aliah Nursyahirah Kamarudin, Zainal Abidin Talib, Hussein Baqiah, Chen Soo Kien, Lim Kean Pah, Muhammad Khalis Abdul Karim, Muhammad Kashfi Shabdin, Abdul Halim Shaari, Azhan Hashim, Nurbaisyatul Ermiza Suhaimi, Muralidhar Miryala

**Affiliations:** 1 Laboratory of Superconductor and Thin Films, Department of Physics, Faculty of Science, Universiti Putra Malaysia, Serdang 43400, Malaysia; 2 Department of Physics, College of Natural Sciences, Jeonbuk National University 567, Baekje-daero, Deokjin-gu, Jeonju-si 54896, Republic of Korea; 3 Shandong Key Laboratory of Biophysics, Institute of Biophysics, Dezhou University, No. 566 University Rd. West, Dezhou 253023, China; 4 Faculty of Applied Sciences, Universiti Teknologi MARA Pahang, Jengka 26400, Malaysia; 5 Materials for Energy and Environmental Laboratory, Superconducting Materials, Shibaura Institute of Technology, 3 Chome-7-5 Toyosu, Koto, Tokyo 135-8548, Japan

**Keywords:** Bi-2223, co-precipitation method, graphene nanoparticles, critical temperature, critical current

## Abstract

The effects of graphene addition on the phase formation and superconducting properties of (Bi_1.6_Pb_0.4_)Sr_2_Ca_2_Cu_3_O_10_ (Bi-2223) ceramics synthesized using the co-precipitation method were systematically investigated. Series samples of Bi-2223 were added with different weight percentages (*x* = 0.0, 0.3, 0.5 and 1.0 wt.%) of graphene nanoparticles. The samples’ phase formations and crystal structures were characterized via X-ray diffraction (XRD), while the superconducting critical temperatures, *T*_c_, were investigated using alternating current susceptibility (ACS). The XRD showed that a high-*T*_c_ phase, Bi-2223, and a small low-*T*_c_ phase, Bi-2212, dominated the samples. The volume fraction of the Bi-2223 phase increased for the sample with x = 0.3 wt.% and 0.5 wt.% of graphene and slightly reduced at x = 1.0 wt.%. The ACS showed that the onset critical temperature, *T*_c-onset_, phase lock-in temperature, *T*_cj,_ and coupling peak temperature, *T*_P_, decreased when graphene was added to the samples. The susceptibility–temperature (χ′-T) and (χ″-T) curves of each sample, where χ′ and χ″ are the real and imaginary parts of the susceptibility, respectively, were obtained. The critical temperature of the pure sample was also measured.

## 1. Introduction

Bismuth strontium calcium copper oxide, BSCCO, has the general chemical formula Bi_2_Sr_2_Ca_n−2_Cu_n_O_x_, and its critical temperatures, *T*_cj_, for the first few members with Bi-2201, Bi-2212, and Bi-2223, are 20 K, 85 K, and 110 K, respectively [1,2,3]. Since Bi-2223 has the highest superconducting transition temperature, *T*_c_, in their series, it is the most attractive for potential application.

BSCCO is a class of high-temperature superconductors (HTS) that has been widely investigated and applied in engineering, medical equipment, mining, and transport systems. In 2004, high-performance and long-length Bi-2223 HTS wires were successfully commercialized and well-received in the market [4,5,6,7,8,9,10]. These commercialized Bi-2223 wires or DI-BSCCO (Dynamically Innovative BSCCO) have been used in several cable projects, such as the Albany project in the United States [6]; the Yokohama project in Japan [7]; the AmpaCity project in Germany [8]; the DC cable project in Saint Petersburg, Russia [9]; and possibly as superconducting feeder cables to railway lines in Japan and France, which is still being appraised [10]. Bulk superconducting materials have various applications, including current leads, electrical fault current limiters, and electromagnetic levitation systems. However, the limitations of the Bi-2223 system, such as weak critical current density, *J*_c_, due to intergrain weak links, and weak flux pinning ability in bulk samples, have hindered progress. The current fabrication technique has succeeded in producing Bi-2223 wires with *J*_c_ as high as 7.4 × 10^4^ A/cm^2^ at 77.3 K and a self-magnetic field [11] for Bi-2223 thin films of 1.3 × 10^6^ A/cm^2^ at 70 K [12]. Magneto-optical analyses have revealed that for Bi-2223 wires, the local *J*_c_ had reached 2.5 × 10^5^ A/cm^2^ [13]. To extend the applications of HTS wires, their basic properties, particularly the performance of *I*_c,_ must be improved to enhance the *J*_c_. One approach that has been broadly accepted is the addition or substitution of various elements into the BSCCO system. Numerous studies have shown that nanoparticles can be easily integrated and spread widely among the grains in the BSCCO system because of their small size. Promising outcomes on the properties of the Bi-2223 system, such as high *T_c_* phase formation, improved intergrain connectivity, flux pinning ability, and critical current density, *J*_c_, were achieved when nanosized dopants were integrated [14,15,16,17,18,19,20].

Graphene, a two-dimensional crystalline carbon material, has been used in many applications, especially electronics, due to its outstanding electrical, mechanical, and chemical properties [21,22]. Graphene is an important carbon-based nanomaterial with outstanding electrical, thermal, and mechanical properties [23,24,25]. Both graphene and Bi-2223 superconductors grow in platelet (sheet)-like microstructures. Therefore, using graphene nanoparticles as an additive in a Bi-2223 system is appealing. Several studies have been conducted on the incorporation of graphene into a superconducting system, such as graphene with yttrium barium copper oxide, YBCO [26,27,28,29,30,31,32,33], magnesium diboride, MgB_2_ [34,35,36], and thallium-based high-temperature superconductors [37]. However, few investigations have been reported for BSCCO systems [38]. Undoubtedly, adding graphene to a superconductor material can have several beneficial effects, including acting as an inter-grain weak link, enhancing the critical current density (*J*_c_), and reducing the average grain diameter.

Recent studies have found that single-phase Bi-2223 superconductors are usually challenging to synthesize because of the complexity of the reaction during phase formation. The co-precipitation method, a wet chemical technique, can often be considered a viable solution for addressing this issue. This is because powders generated through this method possess a reduced grain size, increased purity, and greater homogeneity than those produced using the solid-state method [39,40,41,42,43,44,45,46]. To the best of our knowledge, no work has been carried out on the addition of graphene nanoparticles into Bi-2223 using the wet chemical co-precipitation method. The outcome of this study has the potential to enhance the critical current density (*J*_c_) of Bi-2223 superconductors in future studies. In this paper, we report on the effect of the critical temperature, *T*_c_; the critical current density, *J*_c_; and the structural and morphological properties of bulk Bi_1.6_Pb_0.4_Sr_2_Ca_2_Cu_3_O_y_ when graphene nanoparticles are integrated into the superconductors using the co-precipitation method.

## 2. Materials and Methods

The preparation of Bi_1.6_Pb_0.4_Sr_2_Ca_2_Cu_3_O_y_ was carried out via the co-precipitation method using bismuth(III) acetate (CH_3_COO)_3_Bi (99%), lead(II) acetate (CH_3_COO)_2_Pb·3H_2_O (99%), strontium acetate (CH_3_COO)_2_Sr·0.5H_2_O (98%), calcium acetate (CH_3_COO)_2_Ca·XH_2_O (97%), and copper(II) acetate (CH_3_COO)_2_Cu·H_2_O (98–102%). All chemicals were purchased from Alfa Aesar and used without further purification. The powders were weighed and mixed in a 2:2:2:3 stoichiometric ratio and were dissolved in 500 mL acetate acid and labeled as solution A. Adding lead, Pb, into the BSCCO composition is able to effectively increase the volume fraction of the Bi-2223 phase [47]. Solution B was later prepared by dissolving 25 g of oxalic acid C_2_H_2_O_4_·2H_2_O (99.5–102.5%) in a solution of distilled water:propane-2-ol (1:1.5) to obtain a concentration of 0.5 M. Solution A was stirred at 400 rpm at a temperature of 80 °C, while solution B was stirred at 400 rpm without heating. They were then soaked in an ice bath to achieve a temperature range of 0 °C to 2 °C. Solutions A and B were mixed until a uniform milky blue suspension was obtained. The blue suspension slurry was filtered, dried on a hot plate at 80 °C for 12 h, and ground into powder. The powder was initially calcined in an alumina boat at 730 °C for 12 h to obtain a good texture.

The calcined powders were ground and subjected to another calcination process at 850 °C for 24 h. The initial powder underwent a regrinding process and was combined with graphene sheets obtained from Nanostructured and Amorphous Materials, Inc. in Houston, TX, USA, with an average diameter ranging from 0.5 to 3 µm and a thickness between 0.55 to 1.2 nm. Different weight percentages of graphene sheets were added: 0.0 wt.%, 0.3 wt.%, 0.5 wt.%, and 1.0 wt.%. Subsequently, the mixture was pressed into pellets weighing 1.0 g each using hydraulic press under 5 tons of pressure. The pellets were sintered in a tube furnace at 850 °C for 48 h at a heating rate of 2 °C/min and cooling rate of 1 °C/min. X-ray diffraction (XRD) patterns of the samples were recorded using an X-ray diffraction diffractometer (Xpert Pro Panalytical Philips DY 1861 diffractometer) with CuKα radiation. The thermal material’s stability was monitored using a thermogravimetric analyzer (Mettler Toledo, TGA/SDTA851e), and the measurement of AC magnetic susceptibility as a function of temperature was obtained using an AC Susceptometer (CryoBIND). The morphology and elemental analysis were analyzed using field emission scanning electron microscopy (FESEM, JEOL JSM-7100F). The *J*_c_ of the samples was determined using a four-point probe (4PP) with a 12 K closed-cycle He Cryostat system.

## 3. Results

### 3.1. Thermogravimetric Analysis

**Figure 1:**
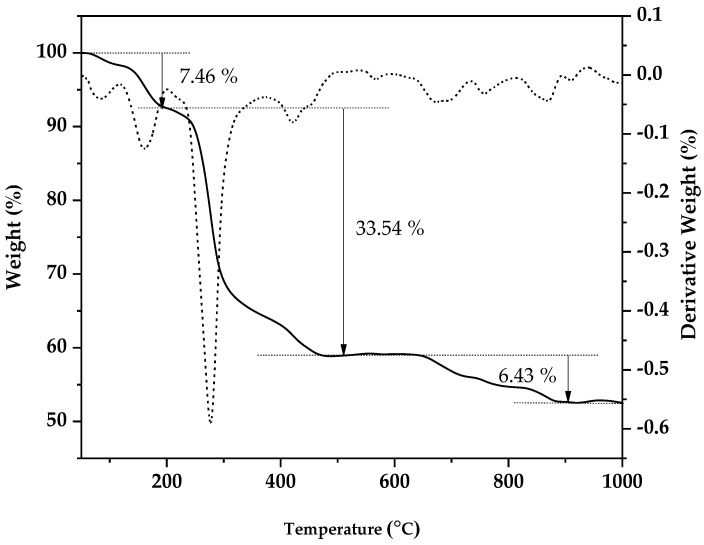
TGA (solid curve) and DTG (dashed curve) for pure Bi-2223 samples before the sintering process.

Thermogravimetric analysis (TGA) and differential thermogravimetry (DTG) curves versus temperature for pure Bi-2223 samples were obtained immediately after the co-precipitation process had been performed and before the sintering process was carried out. The TGA curve of Bi-2223 displayed in Figure 1 shows three different distinctive transitions at various temperatures and the same trend as that in the DTG curves. As the heating rate increased, the peak of the derivative thermogravimetric (DTG) curve shifted toward higher temperatures. The initial phase of the process lead to a 7.46% reduction in weight, which took place within the temperature range of 58.39 to 199.05 °C. This weight loss can be attributed to the evaporation of water and moisture present in the powder. Consequently, the water molecules derived from Bi_2_(C_2_O_4_)_3_, Pb(C_2_O_4_), Sr(C_2_O_4_), Ca(C_2_O_4_), and Cu(C_2_O_4_) contribute to the formation of dehydrated oxalate. The second phase occured between 200.10 °C and 508.82 °C, resulting in a weight loss of 33.54%. This weight reduction is primarily caused by the decomposition of Bi_2_(C_2_O_4_)_3_ into Bi_2_O_3_, Pb(C_2_O_4_) into PbO, Sr(C_2_O_4_) into SrCO_3_, Ca(C_2_O_4_) into CaCO_3_, and Cu(C_2_O_4_) into CuO. Previous studies have also reported similar findings regarding the thermal decomposition of individual oxalates of Bi, Sr, Ca, and Cu. However, for rare earth acetate, the decomposition typically occurs within the temperature range of approximately 200 to 300 °C [45]. The third transition in Figure 1 comprises a weight loss of 6.43% between 536.68 to 932.66 °C, representing the decomposition of CaCO_3_ to CaO and SrCO_3_ to SrO. These results are consistent with that of similar investigations [48]. Figure 1 also shows that the weight losses become almost constant after 850 °C, establishing the optimal temperature for calcination and sintering of BSSCO. This has been confirmed by several researchers reporting that the optimal temperature for the calcination and sintering process for BSSCO is in the range of 840 °C to 850 °C [49,50]. At this temperature, there is also a tendency to crystallize the (Bi,Pb)-2223 from Bi-2212 and other impurities [51].

### 3.2. X-ray Diffraction (XRD) Analysis

**Figure 2:**
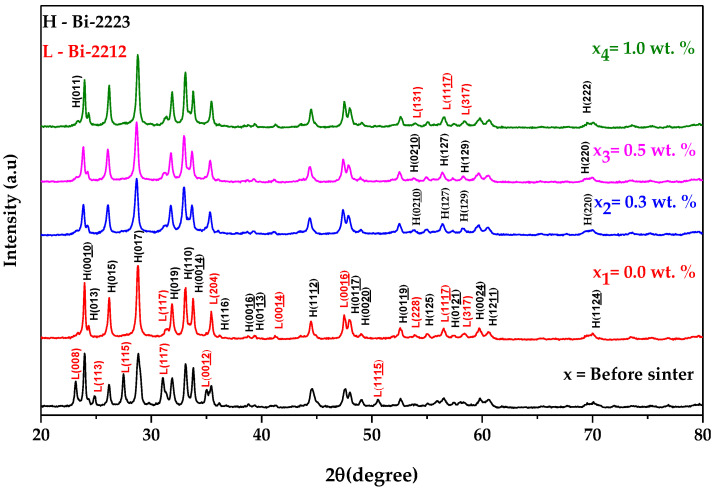
XRD analysis of Bi-2223 samples before and after the sintering process for samples with 0.0 wt.%, 0.3 wt.%, 0.5 wt.%, and 1.0 wt.% addition of graphene. The peak indicated by L and H represents the Bi-2212 and Bi-2223 phases, respectively.

The patterns of XRD for Bi-2223 samples integrated with the graphene nanoparticles of 0, 0.3, 0.5, and 1.0 wt.% are shown in Figure 2. The XRD patterns show that all the secondary phase peaks decreased for the sintered samples. This demonstrates that sintering has successfully reduced the secondary phases in Bi-2223 samples. The XRD peaks for all the samples indexed to Bi-2223 (ICSD: 98-008-0522). The highest intensity of Bi-2223 peaks was observed at 2θ ≈ 28.73°, which matches the Miller indices of the (0 1 7) plane. It can be seen from Figure 2 that the intensity of the peaks decreases when 0.3 wt.% addition of graphene nanoparticles to the samples and increases when 0.5 wt.% and 1.0 wt.% was added. At the same time, the peaks also slightly shifted towards the smaller angle of 2*θ* when the amount of graphene nanoparticles increased in the sample. However, some minor peaks belonging to Bi-2212 (ICSD: 98-003-0405) were also observed in all XRD patterns at 2*θ* ≈ 31.16°, 35.45°, 41.24°, and 47.46°, which indicate the presence of a secondary phase. Unfortunately, no graphene-related peak appears in the XRD results due to the small amount of graphene added. Previous studies have found that the main graphene peak appears around 2*θ* equal to 9.86° [36], 10° [27,33], 12.85° [29], 26.5°, and 54.5° [37]. The number of phases present for Bi-2223 and Bi-2212 was calculated using the following expressions [52,53]:

(1) $$Bi\text{-}2223\left(\%\right)=\frac{\sum I_{2223}}{\sum I_{2223}+\sum I_{2212}}\times 100\%$$

(2) $$Bi\text{-}2212\left(\%\right)=\frac{\sum I_{2212}}{\sum I_{2212}+\sum I_{2223}}\times 100\%$$

where *I* is the peak intensity of the phases. Table 1 shows that the phase of Bi-2223 increases with graphene but declines when 1.0 wt.% graphene nanoparticles are added. A reverse trend was observed for the phase of Bi-2212. The Bi-2223 (0.0 wt.%) XRD pattern shows a tetragonal crystal structure with lattice parameters *a* = *b* = 3.826 Å and *c* = 37.104 Å. All the graphene-added samples given in Table 2 show almost identical lattice parameters, indicating that adding graphene nanoparticles did not distort the Bi-2223 crystal system or become part of the Bi-2223 crystal structure. This indicates that the nanoparticles are located in the middle of superconducting grains. Previous research has indicated that the presence of impurities in nanoparticles can serve in two ways: they can act as pinning centers that immobilize vortices or enhance the interconnection among the grains. This, in turn, can potentially enhance the critical current density *J*_c_ [54,55]. The crystallite size listed in Table 2 was calculated by choosing the highest peak and using the Scherrer equation [56]:

(3) $$L=\frac{K\lambda }{B_{size}\;\cos\theta }$$

where *L* is the crystallite size, *K* equal to 0.9 is a dimensionless shape factor, *B_size_* is the line broadening at half the maximum intensity (FWHM), *λ* equal to 1.5406 Å is the X-ray wavelength for Cu K_α_ radiation, and *θ* is the Bragg angle. Table 2 shows that all the samples possess comparable crystallite sizes. The XRD patterns were also examined using the Williamson–Hall plot method, which also determined the crystallite size and the lattice strain of the sample. Previous studies have established that lattice strain and crystallite size are the primary reasons for the broadening of the diffraction peaks. The Williamson–Hall plot was obtained by utilizing the equation below [57]:

(4) $$\beta_{hkl}\cos\theta =\frac{K\lambda }{L}+4C_{\varepsilon }\;\sin\theta$$

where *C_ε_* is the lattice strain. By plotting *β_hkl_* cos*θ* against 4sin*θ*, the intercept (*Kλ*/*L*) of the plot represents the crystallite size and the gradient represents the lattice strain (*C_ε_*) of the samples [41]. The results shown in Table 2 demonstrate a marked difference between using the Williamson–Hall and Scherrer equations. The finding from the Williamson–Hall plot shows that adding graphene to the samples decreases the crystallite size. Both calculations show that the sample with 0.3 wt.% has the smallest crystallite size. The lattice strains in the sample do not show any noticeable changes after introducing graphene.

### 3.3. Critical Temperature, T_c_ Analysis

**Figure 3:**
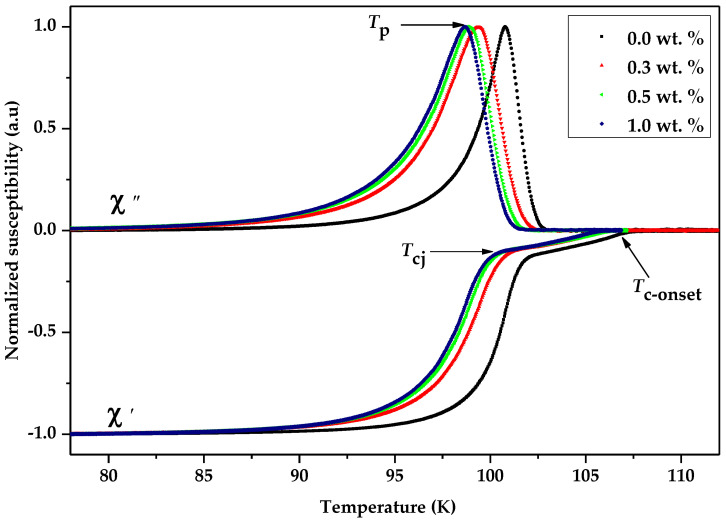
Graph of the normalized susceptibility of the real (χ′) and imaginary part (χ″) against the temperature for samples Bi-2223 with 0.0 wt.%, 0.3 wt.%, 0.5 wt.%, and 1.0 wt.% addition of graphene.

Figure 3 illustrates the temperature dependencies of the real component (χ′) and imaginary component (χ″) of the samples on an applied field of 39.885 A/m and a frequency of 219 Hz, with the field applied parallel to the long dimension of the samples. As seen in the figure, there are two discernible decreases in the real part of AC susceptibility, χ′. The initial decline observed at the onset temperature, *T*_c-onset_, can be attributed to the transition occurring within individual grains (intra-grain). On the other hand, the second decrease observed at the phase-locking temperature, *T*_cj_, results from the superconducting coupling between the grains (inter-grain) [58]. In the temperature range between the onset of superconductivity (*T*_c-onset_) and the critical temperature (*T*_cj_), the superconducting grains are disconnected, making the system resistive. However, below *T*_cj_, the grains become connected and are phase-locked, resulting in zero phase difference across the intragranular junctions. There is a definite transition for *T*_c-onset_ (χ′) to the lower temperature with increasing graphene addition. The decrease in *T*_c-onset_ can be attributed to the reduction in hole concentration resulting from oxygen deficiency in the CuO chain [41]. The findings also show that the intragranular coupling between grains decreased by increasing the wt.% of graphene added to the samples based on the phase-locking temperature, *T*_cj_. Prior work has indicated that the Bi-2212 low-*T*_c_ phase can affect the phase-locking, *T*_cj_ [58].

The peak observed in the imaginary part χ″ represents the measure of dissipation in the samples. The imaginary part of the AC susceptibility (ACS) in all the samples exhibited a single peak at the coupling peak temperature, *T*_p_, corresponding to intergrain coupling. Notably, no secondary peak was observed near the critical temperature, *T*_c_, indicating the absence of an intra-granular peak in any of the samples. This could indicate an excellent quality for the ceramic superconductor in all the samples where the coupling phase occurs immediately after the occurrence of superconducting inside the grain [59]. There could be various reasons for this phenomenon, including inadequate total grain volume and grain size relative to the penetration depth (λ). However, as shown in the table, shifting the *T*_p_ to lower temperatures with the introduction of graphene nanoparticles indicates the weakening of the grains and subsequently reducing the pinning force, while the *T*_p_/*T*_c-onset_ ratio indicates a weak coupling between the grains. The value of Josephson’s current, *I*_o_, was calculated by using the Ambegaokar–Baratoff theory [60,61]:

(5) $$I_{O}=1.57\times 10^{-8}\left(\frac{{T_{c\text{-}onset}}^{2}}{T_{c\text{-}onset}-T_{cj}}\right)$$

Table 3 indicates that *I*_o_ decreases with the increasing amount of graphene nanoparticles in the samples. This may be attributed to an increased presence of unreacted graphene nanoparticles at the boundaries between grains. As a consequence, the tunneling of Josephson’s current across the grains is decreased. In addition, the decreasing *I*_o_ also implies that excess graphene nanoparticles may degrade the grain connectivity.

### 3.4. Critical Current Density, J_c_ Analysis

**Figure 4:**
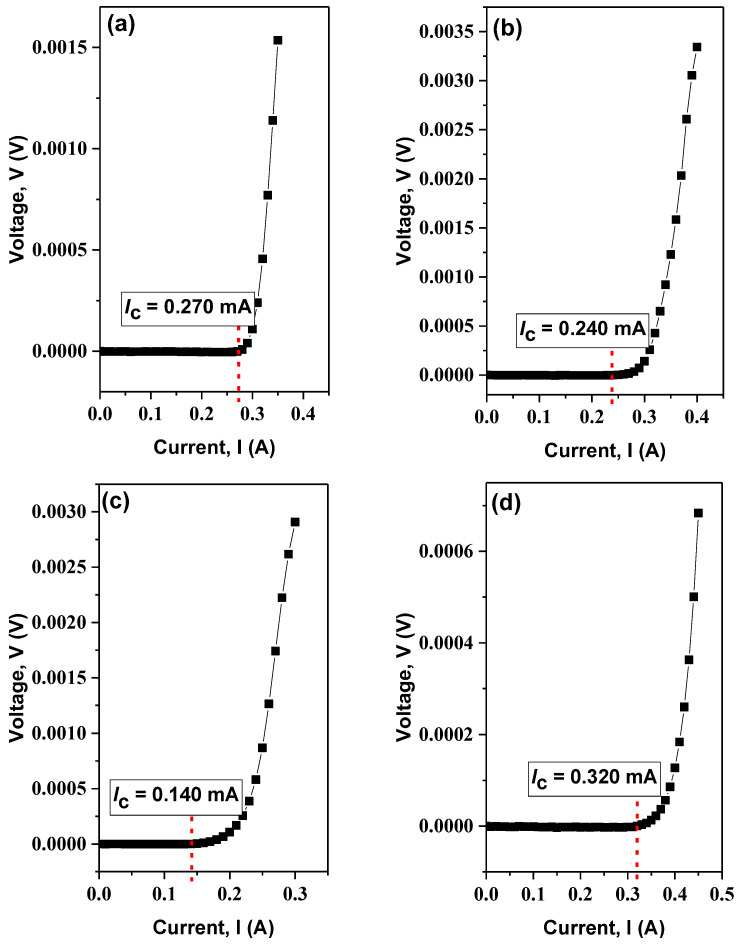
V-I curve in self-field at 40 K for (**a**) x = 0.0 wt.%, (**b**) x = 0.3 wt.%, (**c**) x = 0.5 wt.%, and (**d**) x = 1.0 wt.%.

**Figure 5:**
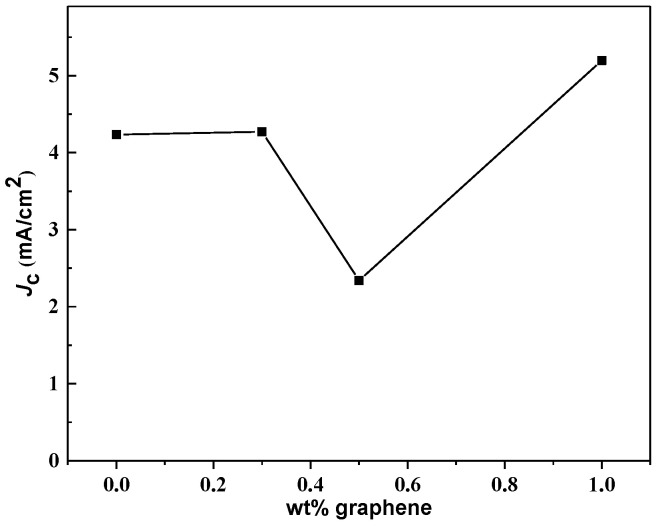
Variation of critical current density, *J*_c_, against weight percentage of graphene addition content.

The graph of voltage, *V* versus current, *I*, shown in Figure 4, displays the critical current, *I*_c_, of the samples measured at 40 K in a zero magnetic field. The transport critical current density, *J*_c_, was calculated using the equation *J*_c_ = *I*_c_/*A* [62], where *A* is the cross-sectional area of the bar-shaped samples. The critical current density increase is observed for samples with 0.3 wt.% and 1.0 wt.% addition of graphene but not for the 0.5 wt.%, as shown in Figure 5. The improvement in *J*_c_ is attributable to improvements in the connectivity of intergrain.

### 3.5. Microstructure Analysis

**Figure 6:**
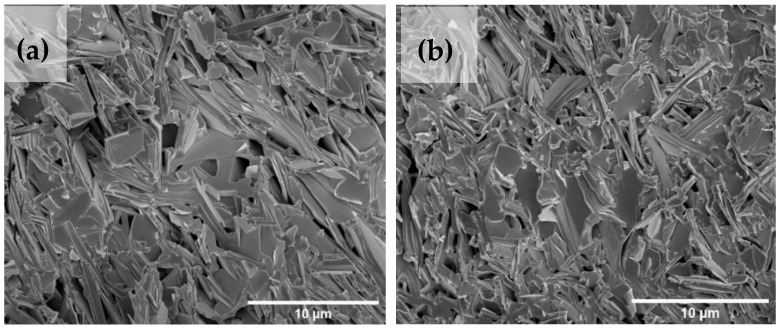

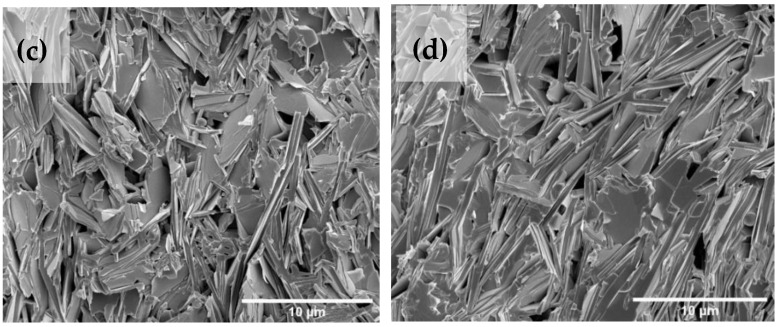
SEM micrographs of Bi-2223 samples with (**a**) x = 0.0 wt.%, (**b**) x = 0.3 wt.%, (**c**) x = 0.5 wt.%, and (**d**) x = 1.0 wt.% of graphene.

**Figure 7:**
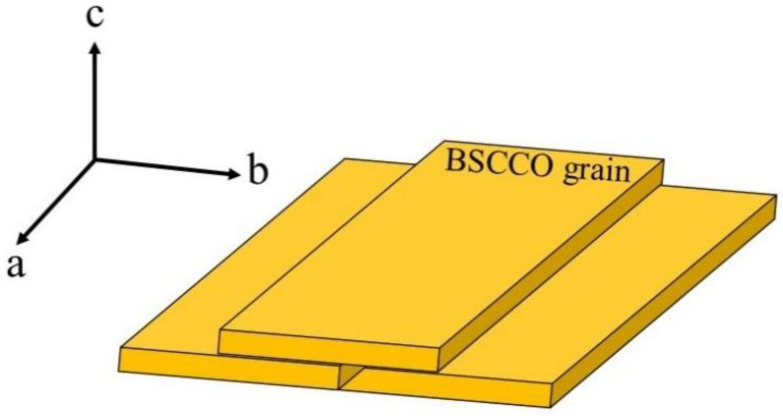
Schematic diagram demonstrating how the BSCCO grain is parallel to its *ab*-plane.

Figure 6 shows the SEM images of the surface morphology of Bi-2223 samples at 10.000X magnification. The microstructures of the samples show varying degrees of alignment of the compacted layers of thin flaky plate-like grains. The grains for samples with graphene are better aligned than those without graphene. The grains for the 1.0 wt.% are mostly aligned parallel to the *ab*-plane compared to other samples. The existence of graphene in Bi-2223 showed the different parts of the structure and distributed uniformly on the grain boundaries in great amounts. According to the analysis depicted in Figure 6, it was observed that the pure sample displayed irregular shapes that were randomly distributed. In contrast, the 1.0 wt.% sample demonstrated a denser grain structure with reduced porosity, indicating a more compacted arrangement. This indicates that the superconducting phase expanded at the expense of the surrounding phase, supporting our explanation of the higher critical temperature (*T*_c_). Figure 7 shows a schematic illustration of how the broad flat surface of the BSCCO grains is always stacking parallel to its *ab*-plane. This observation coincides with the results from four-point probe measurements that the 1.0 wt.% also has the highest critical current density, *J*_c_, as presented in Table 4. The inclusion of graphene nanoparticles resulted in larger grain sizes characterized by a tightly packed arrangement. This facilitates grain growth and plays a role in enhancing intergranular transport currents [26]. Possible explanations for the current transport of the grain morphology of Bi-2223 tapes can be found in the brick wall (BW) model [63,64] and railway switch (RS) model [65,66], which explain the behavior of c-axis [001] twist grain boundaries (GBs) in Bi-2223 samples. According to the BW model, the c-axis GBs create a highly connected supercurrent path when the *ab*-plane is blocked. This occurs due to the large surface area of basal-plane-faced GBs, which are formed by high aspect ratio grains. This configuration allows for a strong linkage of c-axis supercurrents. In contrast, the RS model proposes a different mechanism. It suggests that the *ab*-plane supercurrent path is primarily established through low-angle, strongly coupled, and obliquely intersecting GBs called small-angle, c-axis tilt (SCTILT) GBs. These GBs contribute to improving the texture of the samples. The presence of these well-defined supercurrent paths in both models improves the transport current occupancy of the Bi-2223 tape’s cross-section. As a result, the need to rely on intrinsically lower critical current density (*J*_c_) c-axis paths between dominant *ab*-plane paths is reduced. Ultimately, this improved connectivity and transport current occupancy enhance the *J*_c_ of the Bi-2223 tapes [67,68].

Data presented in Table 5 from EDX analysis indicate that the atomic ratio of Bi, Sr, Ca, and Cu is 2:2:2:3, with different oxygen ratios obtained for all samples. This confirms that all samples have Bi-2223 element composition, which supports the XRD results of all peaks indexed to Bi-2223 phases. The atomic percentage for C also increases, proving that graphene was added to the samples, as shown in Table 6. However, it can be seen that increasing graphene addition showed the results of unreacted graphene and localized inhomogeneity within the samples.

**Figure 8:**
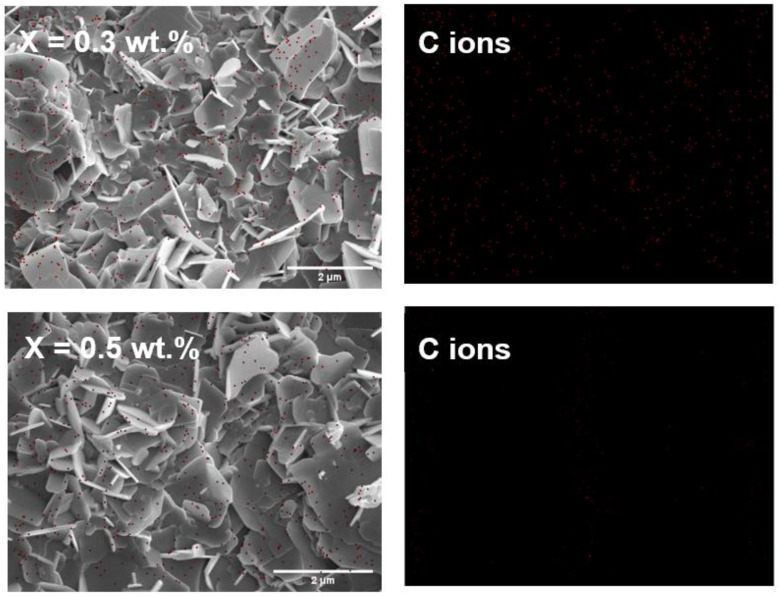

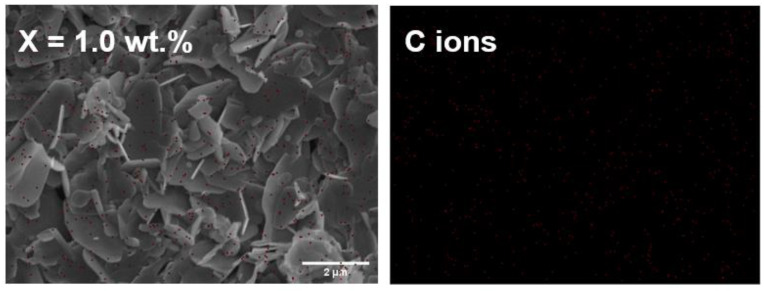
FESEM images and EDX mapping C ions (red dots) of the compound Bi-2223 with added graphene nanoparticles of up to x = 0.3 wt.%, 0.5 wt.%, and 1.0 wt.%, indicating the presence of the expected elements.

The information provided suggests that the elements of carbon were detected in all samples with a percentage range of 7.57% to 10.54%. The presence of graphene (C) in Bi-2223 was confirmed through elemental mapping using energy dispersive X-ray spectroscopy (EDX) as shown in Figure 8 for samples with different graphene concentrations (x = 0.3 wt.%, 0.5 wt.%, and 1.0 wt.%). This verifies the existence of carbon, which was not observed in the X-ray diffraction (XRD) patterns. Furthermore, the atomic percentage of carbon elements increased from 32.07% to 39.29% as the graphene nanoparticle content increased from x = 0.3 wt.% to x = 1.0 wt.% in the Bi-2223 samples. This finding supports a previous study that demonstrated increased graphene content with increasing graphene nanoparticle concentration in Bi-2223 samples [69].

## 4. Conclusions

Bi_1.6_Pb_0.4_Sr_2_Ca_2_Cu_3_O_y_ superconducting samples with the addition of graphene nanoparticles at weight percentages of 0.0, 0.3, 0.5, and 1.0 wt.% were successfully synthesized using the co-precipitation method. A variation in *T*_c_ and *J*_c_ values was observed due to the addition of graphene content in Bi-2223. Elemental analysis via EDX showed that stoichiometry ratio of Bi-2223 superconducting compounds were obtained. The XRD patterns exhibited the primary phase indexed to Bi-2223, corresponding to a tetragonal structure, and a second phase which belonged to Bi-2212. ACS measurements established that the onset critical temperature, *T*_c-onset_; coupling peak temperature, *T*_p_; and intergranular critical current density, *T*_cj_, decreased with the increase in graphene nanoparticle addition. The decrease in the Josephson current, *I*_o_, with the increased addition of graphene was due to the degradation of samples’ grain connection due to the amount of unreacted graphene at the grain boundary increase in the samples. The sample with 1.0 wt.% of graphene produced the highest *J*_c_ of all the samples. SEM results proved that the microstructure for samples with 1.0 wt.% was the best aligned and the grain showed a much better formation of Bi-2223 superconductors. Based on these results, adding graphene nanoparticles to Bi-2223 samples could be able to improve the pinning center and hence enhanced the critical current density, *J*_c_.

## Figures and Tables

**Table 1:** The percentage of Bi-2212 phase formed in Bi-2223 with 0.0 wt.%, 0.3 wt.%, 0.5 wt.%, and 1.0 wt.% addition of graphene.

Bi-2223 + x wt.% of Graphene	Intensity Fraction (%)	
	Bi-2223	Bi-2212
0.0	85.17	14.83
0.3	87.43	12.57
0.5	88.25	11.75
1.0	84.00	16.00

**Table 2:** The percentage of Bi-2212 phase formed in Bi-2223 with 0.0 wt.%, 0.3 wt.%, 0.5 wt.%, and 1.0 wt.% addition of graphene.

Bi-2223 + x wt.% of Graphene	Lattice Parameter (Å)			Crystallite Size (nm)		Unit Cell Volume (Å^3^)	Lattice Strain (%)
	*a*-Axis	*b*-Axis	*c*-Axis	Scherrer	Williamson–Hall		
0.0	3.826 ± 0.000659	3.826 ± 0.000659	37.104 ± 0.008048	46.26	397	543.083	0.175
0.3	3.824 ± 0.000760	3.824 ± 0.000760	37.064 ± 0.009404	39.09	74.5	541.859	0.070
0.5	3.823 ± 0.000703	3.823± 0.000703	37.062 ± 0.008636	46.25	182	541.768	0.179
1.0	3.823 ± 0.000635	3.823 ± 0.000635	37.066 ± 0.007766	46.26	109	541.791	0.073

**Table 3:** Coupling peak temperature, *T*_p_, onset critical temperature, *T*_c-onset_, phase lock-in temperature, *T*_cj_, and Josephson current, *I*_o_ for Bi-2223 with 0.0 wt.%, 0.3 wt.%, 0.5 wt.%, and 1.0 wt.% addition of graphene.

Bi-2223 + x wt.% of Graphene	*T*_p_ (K)	*T*_c-onset_ (K)	*T*_p_/*T*_c-onset_	*T*_cj_ (K)	*I*_o_ (µA)
0.0	100.78	107.69	0.936	101.91	31.50
0.3	99.10	107.21	0.924	100.94	28.80
0.5	98.92	106.60	0.928	100.23	28.01
1.0	98.78	106.54	0.927	99.90	26.84

**Table 4:** Critical current density, *J*_c_, of Bi-2223 with the addition of graphene 0.0 wt.%, 0.3 wt.%, 0.5 wt.%, and 1.0 wt.% samples.

Bi-2223 + x wt.% of Graphene	*I*_c_ (A)	*J*_c_ (mA/cm^2^)
0.0	0.270	4235
0.3	0.240	4271
0.5	0.140	2340
1.0	0.320	5197

**Table 5:** EDX analysis of the atomic percentage (at.%) for Bi-2223 with the addition of the graphene of 0.0 wt.%, 0.3 wt.%, 0.5 wt.%, and 1.0 wt.%.

Bi-2223 + x wt.% of Graphene (wt.%)	Elements (%)						
	Bi	Pb	Sr	Ca	Cu	O	C
0.0 (2.0:0.8:2.2:2.0:3.5:6.3)	11.98	4.86	12.93	12.11	20.79	37.33	-
0.3 (2.0:0.3:1.8:1.7:3.5:5.4)	9.23	1.39	8.53	7.68	16.34	24.77	32.07
0.5 (2.0:0.4:2.0:1.7:3.5:6.0)	8.51	1.54	8.31	7.41	14.87	25.71	33.66
1.0 (2.0:0.4:2.0:1.8:3.4:6.6)	7.5	1.52	7.31	6.71	12.84	24.83	39.29

**Table 6:** EDX analysis of the weight percentage (wt.%) for Bi-2223 with the addition of the graphene of 0.0 wt.%, 0.3 wt.%, 0.5 wt.%, and 1.0 wt.%.

Bi-2223 + x wt.% of Graphene (wt.%)	Elements (%)						
	Bi	Pb	Sr	Ca	Cu	O	C
0.0	35.54	14.28	16.08	6.89	18.75	8.48	-
0.3	37.90	5.64	14.68	6.04	20.39	7.78	7.57
0.5	36.43	6.52	14.91	6.08	19.35	8.42	8.28
1.0	35.02	7.04	14.30	6.01	18.22	8.87	10.54

## Data Availability

The data presented in this study are available on request from the corresponding author. The data are not publicly available due to ethical restrictions.
